# Identification of clusters of rapid and slow decliners among subjects at risk for Alzheimer’s disease

**DOI:** 10.1038/s41598-017-06624-y

**Published:** 2017-07-28

**Authors:** Dragan Gamberger, Nada Lavrač, Shantanu Srivatsa, Rudolph E. Tanzi, P. Murali Doraiswamy

**Affiliations:** 1 Ruđer Bošković Institute, Zagreb, Croatia; 2 Jožef Stefan Institute, Ljubljana, Slovenia; 3 Duke Institute for Brain Sciences, Duke University Health System, Durham, USA; 4 Genetics and Aging Research Unit and Department of Neurology, Massachusetts General Hospital and Harvard Medical School, Boston, USA; 5 Neurocognitive Disorders Program, Division of Translational Neuroscience, Department of Psychiatry, Duke University Health System, Durham, USA

## Abstract

The heterogeneity of Alzheimer’s disease contributes to the high failure rate of prior clinical trials. We analyzed 5-year longitudinal outcomes and biomarker data from 562 subjects with mild cognitive impairment (MCI) from two national studies (ADNI) using a novel multilayer clustering algorithm. The algorithm identified homogenous clusters of MCI subjects with markedly different prognostic cognitive trajectories. A cluster of 240 rapid decliners had 2-fold greater atrophy and progressed to dementia at almost 5 times the rate of a cluster of 184 slow decliners. A classifier for identifying rapid decliners in one study showed high sensitivity and specificity in the second study. Characterizing subgroups of at risk subjects, with diverse prognostic outcomes, may provide novel mechanistic insights and facilitate clinical trials of drugs to delay the onset of AD.

## Introduction

Alzheimer’s disease is a major public health concern worldwide and the leading cause of dementia in late life. There are no therapies to slow progression or delay its onset. Consequently, there is an urgent need to develop accurate prognostic tests and effective disease modifying therapies. The 99% failure rate of clinical drug trials over the past two decades^1^ points to both our incomplete knowledge of pathology and prognostics. Both clinical experience and research outcome study data have shown that AD is a heterogeneous condition with high individual variability in age of onset, rate of clinical decline as well as degree of underlying pathology^2–5^. Characterizing subgroups of at risk subjects, with homogenous but diverse prognostic outcomes, may provide novel mechanistic insights and facilitate clinical trials of drug to delay AD onset.

Of the nearly 5 million people affected by AD dementia in the US, it has been estimated that 60% are women. In addition to individual heterogeneity, the study of potential sex differences in AD epidemiology, biology and therapeutics has been a relatively neglected area of research (reviewed in refs 6 and 7. The reported higher prevalence of Alzheimer’s disease (AD) in women had been attributed previously to longer female life expectancy or a detection bias but some, but not all, recent findings suggest that older women may be at greater risk for AD than men^6, 7^. For example, one study found that the age-specific risk of AD was almost two-fold greater in women than men (17.2% versus 9.1% at age 65 years and 28.5% versus 10.2% at age 75 years)^8^ and some other studies find that sex-differences become most prominent among eighty year olds^7^. Potential mechanisms to explain such differences include greater effects of the Apolipoprotein E4 allele in women, sex hormones (such as estrogen), lower cognitive reserve, and differences in occupational or educational attainment (reviewed in refs 6 and 7. Sex differences in immune system responsivity, MRI brain atrophy rates^9^ and effects of plaque-tangle pathology^10^ have also been reported. In contrast, other studies report a higher risk for men to develop verbal memory loss, incident MCI^6^ and cerebrovascular disease^6^. Overall these studies argue for a more definitive examination of sex differences in the vulnerability to AD.

AD may have a prolonged preclinical and prodromal phase and there is great interest in characterizing these phases using biomarkers. Mild cognitive impairment (MCI) is a risk factor for AD and is clinically characterized by mild cognitive deficits but relatively normal everyday functioning and the absence of dementia^11, 12^. Prior studies have documented that MCI subjects have an intermediate phenotype between AD and cognitively healthy subjects with regards to cognition, hippocampal atrophy, neuronal metabolism and cortical fibrillary amyloid pathology (determined) (reviewed in refs 11 and 12. While MCI has been divided into amnestic, non-amnestic and multi-domain MCI, even amnestic MCI is not homogenous^13^. Approximately 10–15% of such subjects may progress to dementia on an annualized basis but there is considerable variability from study to study and within the group – many MCI subjects remain cognitively stable for years and some even revert to normal cognitive states. Thus, identifying subgroups within MCI remains a priority^13^. A number of baseline factors have been linked to such variability. For example, in one 36-month study, the annualized rate of conversion from amnestic MCI to AD dementia was higher in amyloid-positive versus amyloid negative MCI subjects^14^. Such results have led to attempts to further subgroup amnestic MCI based on pathological or neuronal loss biomarkers to improve the homogeneity and accuracy of predicting prognostic outcome^15^. While studies have shown that combining multiple baseline markers does improve prediction, there is no consensus on the best combination of predictive markers and no biomarker has been fully validated and approved for predicting future dementia risk.

These findings are not surprising due to a high degree of randomness in the MCI data as a consequence of the fact that cognitive impairment can have different causes and different manifestations and be affected by multiple biological and measurement factors. For such a relatively noisy domain it is normal that the detected biomarker and clinical prognostic correlations (using traditional statistical approaches) are weak or only moderately strong.

Unsupervised cluster analysis is a data mining method that is increasingly used across many diverse fields to unearth new insights in multidimensional data. It does not require explicit assumptions about the target variables. Such methods may also offer insights into AD given the variability in clinical outcomes and prior autopsy literature noting the existence of patient clusters with unique pathological phenotypes (such as a subgroup with very localized cortical distribution of senile plaques versus another subgroup with more widely distributed plaques)^16^. To our knowledge, such clustering algorithms have not been previously applied to the study of longitudinal changes in people at risk for AD.

The development of an interactive data mining multilayer clustering algorithm (MLC) has been spurred, in part, by recently introduced approaches of redescription mining^17^ and multi-view learning^18^. MLC enables the size and the number of clusters to be determined automatically. The algorithm consists of two steps; in the first step an example similarity table (EST) is computed for each data layer and in the second step these tables are used by an agglomerative bottom-up procedure to find an optimal clustering solution. Similarity of instances is determined by execution of a supervised machine learning algorithm on an artificial classification task which is formulated so that original instances are positive class examples while randomized original instances are in the negative class^19^. The supervised learning algorithm constructs rules that can discriminate between original examples and randomized examples^20^. Multilayer clustering results in improved quality over single layer clustering methods, does not require statistical independence of input data layers, and requires no explicit definition of the distance measure among instances (patients) or the number or size of the resulting clusters. The quality of the obtained clusters can then be reviewed by a dementia domain expert for clinical significance.

The aim of this study was to apply a novel multi-layer clustering algorithm to a longitudinal cohort of MCI subjects to discover homogenous clusters based on baseline and prognostic characteristics. A secondary aim was to test for sex differences within clusters. We studied a large well characterized cohort of late amnestic MCI subjects who were recruited for two multicenter Alzheimer’s Disease Neuroimaging Initiative studies (ADNI-1, ADNI-2) and followed for up to 5 years longitudinally with clinical, cognitive and biomarker (volumetric brain MRI, amyloid PET, FDG-PET, spinal fluid) tests.

## Results

We studied 562 MCI subjects comprising 218 women and 344 men. The mean (SD) age for all subjects was 74.0 (7.5) years and 54.3% were positive for the ApoE4 genotype. Table 1 depicts characteristics of the overall group as well as male and female MCI subjects.

**Table 1:** Baseline Demographic and Clinical Variables.

	MCI Mean (SD)	Female MCI Mean (SD)	Male MCI Mean (SD)	Significance
N	562	218	344	—
Age	74.0 (7.5)	72.8 (7.6)	74.8 (7.3)	<0.01
Education	15.9 (2.9)	15.4 (2.8)	16.2 (3.0)	<0.01
CDR-SB	1.6 (0.9)	1.6 (0.9)	1.6 (1.0)	—
ADAS-11	11.5 (4.6)	11.2 (4.8)	11.8 (4.4)	—
MMSE	27.2 (1.8)	27.1 (1.8)	27.2 (1.8)	—
RAVLT-immediate	31.3 (9.5)	33.9 (10.6)	29.7 (8.4)	<0.001
APOE4 + (%)	54%	58%	52%	—
F/U (months)	34.2 (14.2)	34.3 (14.1)	34.1 (14.3)	—

ADAS = Alzheimer’s Disease Assessment Scale Cognitive Subscale Total Score; CDR-SB = Clinical dementia Rating Sum of Boxes; MMSE = Mini-Mental State Exam total score; RAVLT = Rey Auditory Verbal Learning Test; F/u = follow up duration averaged between the two studies; P-values are for comparison of male versus female subjects.

### Correlation Network Among Baseline and Longitudinal Clinical and Biomarker Variables

**Figure 1:**
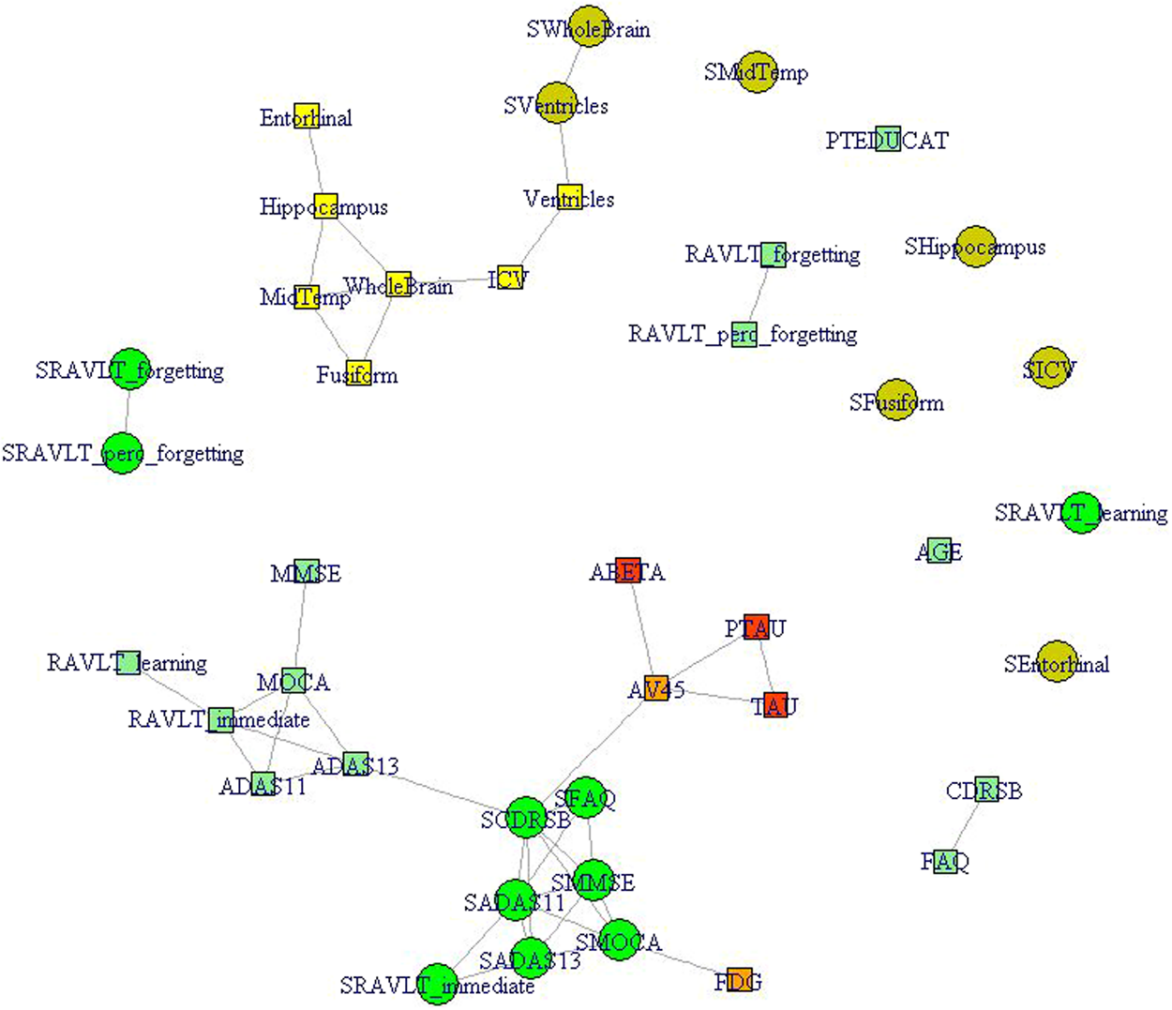
Correlation Network between clinical and biomarker variables in all MCI subjects. The network Baseline descriptors are denoted by squares while circles are used to denote longitudinal rate of change (slope) descriptors. Green color depicts cognitive and functional variables with squares depicting baseline values and circles depicting rate of change values. Yellow squares depict baseline MRI volumetric measures while yellow circles depict slope of MRI volumetric changes over time. Orange squares represent baseline brain FDG-PET and AV45PET data while red squares represent baseline spinal fluid amyloid-beta, total-tau and phosphorylated-tau data. See Methods for details of the measurements. The length of the edges is inversely proportional to the strength of the correlation. Only Spearman’s correlations *rho* > *0.5* are shown as edges. Slopes are denoted with the prefix alphabet “S” in front of the test name. FAQ = Functional Activities Questionnaire; ADAS11 and ADAS13 reflect the 11-item and 13-item versions of this test. MOCA = Montreal Cognitive Assessment Scale; Other details are described in the Methods.

We performed a Fruchterman-Reingold force-directed correlation network graph for the entire sample as a first step to examine inter-relationships between various baseline and longitudinal rate of change variables (Fig. 1). Each variable is a node and the edges represent correlations with shorter edges representing stronger correlations.

One observation is that there are many unconnected nodes (like Age and Education) and some locally connected groups like baseline volumetric descriptors (yellow squares). The largest connected subnetwork is concentrated around the longitudinal slopes of cognitive and functional clinical scales (dark green circles denoting slopes of ADAS-Cog13, CDR-SB, MMSE, FAQ). On one side of these prognostic clinical descriptors are corresponding baseline clinical descriptors (light green squares presenting for example baseline ADAS-Cog13, MOCA, etc). On another side is baseline FDG-PET and yet another side is baseline pathological biomarkers such as AV45 PET and spinal fluid biomarkers (Aβ42, total-tau, p-tau). And as expected, AV45 PET is linked to CSF amyloid-beta and tau measures. Also the two baseline functional measures, CDR-SB and FAQ, are tight linked.

The network also depicts clearly that baseline pathological biomarkers are better correlated with longitudinal slopes of clinical and cognitive tests than they are with the baseline cognitive or functional tests. This is more explicitly demonstrated in Table 2 which compares correlations between biomarker data (spinal fluid and PET data) with baseline values versus longitudinal rate of change (slope) values of key cognitive and functional tests (ADAS-Cog13, CDR-SB and MMSE). For example, the correlation between baseline PET AV45 and the rate of change of CDR-SB is twice that of the correlation between PET AV45 and baseline CDR-SB.

**Table 2:** Correlations between biomarkers and clinical data for all MCI subjects.

	ADASCOG 13 Baseline Slope		CDR-SB Baseline Slope		MMSE Baseline Slope	
**Pathological Markers**						
**Aβ42**	−0.24	−0.38	−0.16	−0.40	0.20	0.36
**T-TAU**	0.29	0.31	0.18	0.34	−0.15	−0.35
**P-TAU**	0.33	0.34	0.17	0.34	−0.12	−0.33
**18F-AV45 PET**	0.39	0.49	0.28	0.58	−0.32	−0.50
**Neural Metabolism**						
**18F-FDG PET**	−0.38	−0.38	−0.25	−0.45	0.30	0.42

Baseline = baseline clinical data; Slope = rate of change of clinical data. The correlations between biomarker data and baseline clinical data are weaker than those with the rate of change of clinical data.

### Identification of homogeneous MCI subpopulations

A clustering tool which implements the multi-layer clustering algorithm was used to construct clusters of MCI subjects. Data on all clinical, MRI, PET and CSF biomarkers were used to determine the similarity among subjects. In the first data layer have been 26 baseline descriptors while 17 prognostic descriptors have been in the second data layer.

**Figure 2:**
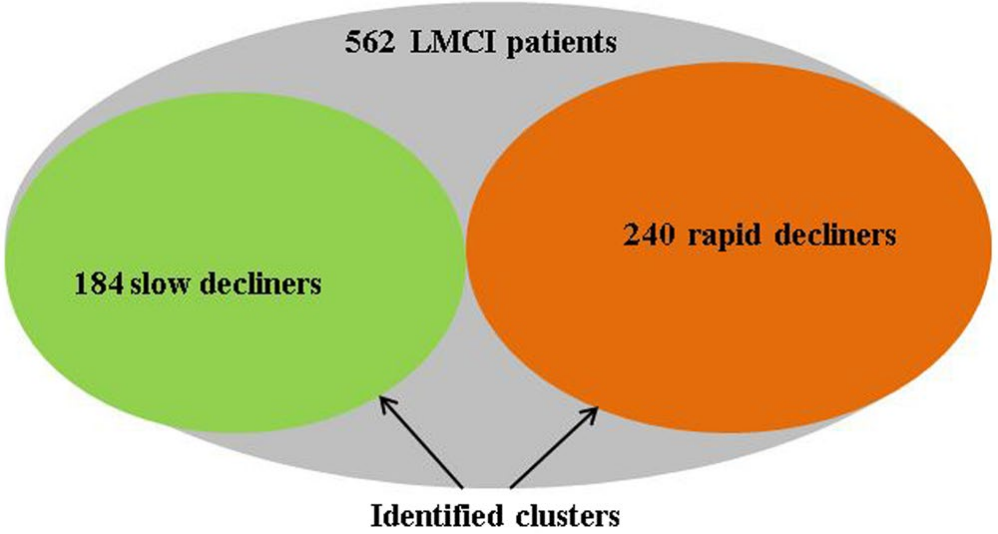
Clustering of the total (N = 562) MCI sample into Rapid and Slow decliners.

Figure 2 graphically illustrates that our clustering method identified two clusters of subjects termed as “*Slow decliners*” and “*Rapid decliners*”. Slow decliners (N = 184) include a subset of MCI subjects that have both favorable baseline data and prognosis while rapid decliners (N = 240) consists of a subset of MCI subjects with a more impaired baseline status and a rapidly progressing longitudinal cognitive course. Our method also identified another 138 patients who did not fit into either cluster - many with baseline ADAS scores in-between slow and rapid decliners.

Table 3 presents differences between the clusters of slow and rapid decliners in their baseline characteristics as well as longitudinal slopes of change for specific cognitive and functional variables. The Mann-Whitney test was used to determine statistical significance of numerical properties and chi-square test has been used for the presence of at least one APOE4+ modification. As shown in Table 3, rapid decliners were more likely to be ApoE4+ and had slightly lower educational levels. Rapid decliners also had substantially worse baseline cognitive and functional measures as well as larger ventricles and smaller medial temporal lobe brain volumes than slow decliners. Rapid decliners also had lower CSF amyloid-beta42 and higher tau levels than slow decliners. Rapid decliners declined at much faster rates than slow decliners on most cognitive and functional measures and had faster rates of brain atrophy. For example, baseline ADAS13 average values are 11.7 and 24.5 for slow and rapid decliners, respectively, a two-fold difference. Likewise, the value of annualized decline for the ADAS-Cog13 is 2.5 times higher in rapid decliners versus slow decliners, and that for the CDR-SB is 7 times greater. A similar effect is present for some objectively measured data like MRI volume of lateral ventricles. Thus, although both groups were classified as late-MCI, these two subpopulations are substantially different.

**Table 3:** Clinical and Biomarker Differences between slow and rapid decliners

	Slow decliners Mean (SD)	Rapid decliners Mean (SD)	z-score
**Baseline Clinical Variables**	N = 184	N = 240	
Education	16.6 (2.6)	15.4 (3.1)	3.8
APOE4+(%)	49%	77%	**
MMSE	28.1 (1.6)	26.4 (1.7)	9.4
ADASCOG-13	11.7 (3.3)	24.5 (4.2)	17.6
MOCA	24.7 (2.9)	20.6 (2.4)	7.1
RAVLT-%forgetting	45.8 (30.4)	83.7 (23.7)	11.7
FAQ	2.2 (3.7)	5.3 (4.9)	7.8
CDR-SB	1.4 (0.8)	2.0 (1.0)	6.7
**Baseline MRI, PET and CSF Variables**			
MRI Ventricles	38 809 (21 120)	46 461 (24 608)	3.4
MRI Hippocampus	6 905 (1 055)	6 096 (964)	7.0
Entorhinal Cortex	3 681 (746)	3 055 (656)	7.8
MRI Medial Temp	19 716 (2 670)	18 348 (2 970)	4.4
Aβ42	177 (56)	148 (45)	4.1
T-TAU	80 (41)	121 (67)	5.4
P-TAU	32 (18)	48 (24)	5.8
FDG PET SUVR	1.26 (0.13)	1.16 (0.11)	6.1
AV45 PET SUVR	1.18 (0.18)	1.38 (0.24)	4.5
**Longitudinal Clinical Slopes**			
SMMSE	−0.1 (0.8)	−1.0 (1.3)	9.9
SMOCA	−0.2 (0.9)	−0.7 (0.9)	3.5
SADASCOG13	0.7 (2.0)	1.9 (2.6)	6.9
SFAQ	0.5 (1.3)	1.7 (2.4)	8.8
SCDR-SB	0.1 (0.4)	0.7 (0.8)	11.6
**Longitudinal MRI Slopes**			
SVentricles	1128 (1079)	2197 (1801)	7.5
SHippocampus	−64 (75)	−113 (110)	6.7
SWholeBrain	−3600 (6000)	−7600 (8100)	6.3
SEntorhinal Cortex	−33 (176)	−71 (187)	3.4
SFusiform	−102 (308)	−282 (391)	6.2
SMedial Temp	−138 (287)	−344 (449)	6.0

All variables in the table were statistically significant at least p < 0.01 or greater using Mann Whitney test. ApoE4 prevalence was tested using the chi-square test and was also significant between groups. The prefix “S” depicts longitudinal change slope. See statistical methods for details.

**Figure 3:**
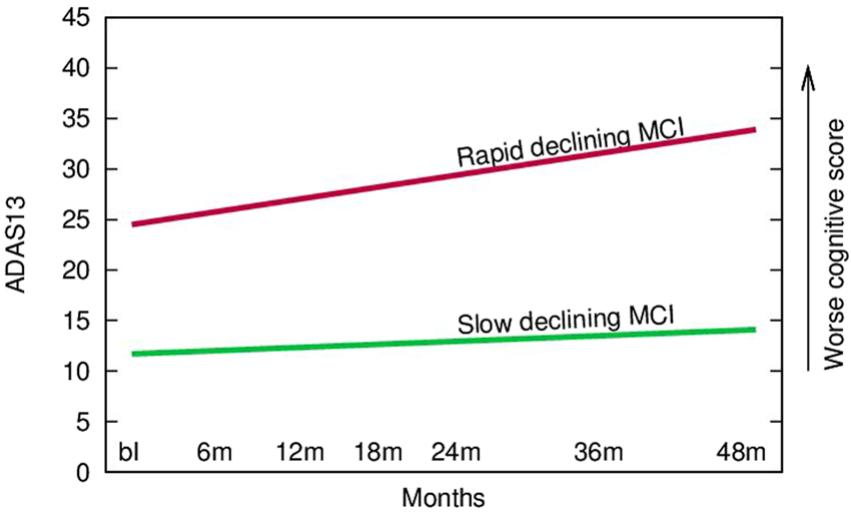
X-axis depicts duration of follow up. Y-axis depicts ADAS-Cog-13 total scores and higher scores depict greater worsening of cognition (due to more cognitive errors). The slopes depict the markedly different cognitive baseline and endpoint for slow versus rapid declining subpopulations of MCI subjects.

Figure 3 depicts differences in the ADAS-Cog 13 between slow and rapid decliners to illustrate the substantial differences in both baseline value and slope of change.

### Progression from MCI to Dementia in Slow versus Rapid Subpopulations

We examined the categorical diagnostic changes from MCI to dementia as determined by the clinician. At each visit, the diagnosis of the subject as reassessed by the site physician using all available information and the clinician rated whether the subject was stable, had converted to dementia or reverted to cognitively normal status. For the complete MCI population the rate of conversion to dementia from MCI was 42% and the rate of reversion from MCI to normal was 4%. The rate of conversion to dementia from MCI was 64% in the rapid cluster and 13% in the slow cluster. In the rapid cluster there was no reversion from MCI to normal while in the slow cluster it has been 10%. These differences were statistically significant (p < 0.001).

### Classifiers to Identify Rapid Decliners

Subgroup discovery technique was used to identify the best classifiers (clinical test cut-offs on ADAS, MMSE and RAVLT) to identify MCI rapid decliners. The classifiers were first developed using ADNI-1 study MCI data and then replicated and validated using the ADNI-2 study MCI data. Table 3 presents sensitivity and specificity of the classifiers. All MCI subjects were included and unclassified patients were combined with slow decliners for this analysis.

A baseline ADAS13 > 19.50 yielded a sensitivity and specificity in ADNI1 of 92.0% and 93.7% respectively, and was also highly accurate in ADNI2 with sensitivity and specificity of 98.4% and 90.0%. A baseline cut-off on ADAS11 > 12.0 yielded satisfactory metrics in ADNI1 of 80.7% and 93.7% versus in ADNI2 of 89.1% and 98.0%. In total 230 rapid decliners (95.8%) satisfy at least one of these conditions and 194 of them (80.8%) satisfy both conditions. Using combined cut-off scores from ADAS11 and ADAS13 yielded the highest accuracy (Table 4). An MMSE cut-off < 27 yielded the worst sensitivity and specificity of 56.8% and 74.3% in ADNI1 and 46.9% and 79% in ADNI2. The RAVLT-immediate < 30 yielded intermediate accuracy.

**Table 4:** Sensitivity and specificity of classifiers for identifying Rapid Declining MCI.

	ADNI1		ADNI2	
	Sensitivity	Specificity	Sensitivity	Specificity
ADAS13 > 19.50	92.0%	93.7%	98.4%	90.0%
ADAS11 > 12.0	80.7%	93.7%	89.1%	98.0%
RAVLT_immediate < 30	75.6%	73.0%	75.0%	70.0%
ADAS11 > 10.5 AND ADAS13 > 19.0	93.8%	95.5%	98.4%	94.0%

### Effect of Gender on Rapid versus Slow Decliner Status

**Figure 4:**
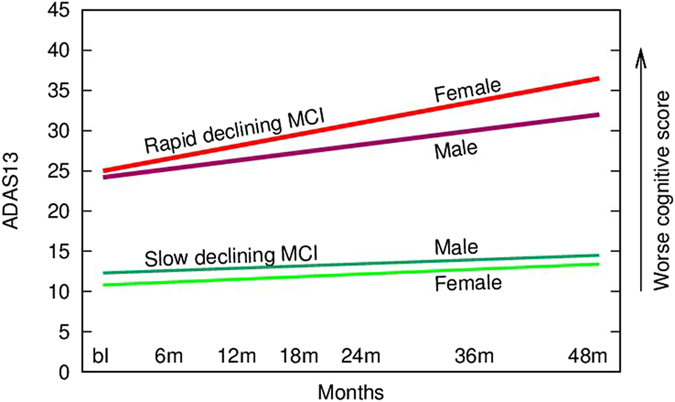
X-axis depicts duration of follow up. Y-axis depicts ADAS-Cog-13 total scores and higher scores depict greater worsening of cognition (due to more cognitive errors). The slopes depict sex-specific cognitive baseline and endpoint scores for slow versus rapid declining subpopulations of MCI subjects.

At entry, men were slightly older, better educated, had larger brain volumes (*p* < 0.*001*) and lower RAVLT immediate recall score (*p* < 0.*001*) than women (Table 1). In both men and women, rapid decliners had worse baseline cognitive status, smaller brain volumes, FDG-PET hypometabolism, higher amyloid and tau markers and more rapid atrophy than slow decliners (Table 5, Fig. 4). Among women, the rate of conversion to dementia from MCI was 69% in the rapid cluster and 9% in the slow cluster whereas among males this was 61% and 16% respectively.

**Table 5:** Sex-specific clinical and biomarker differences between MCI Clusters.

	Females			Males		
	Slow decliners mean (SD)	Rapid decliners mean (SD)	Sig.	Slow decliners mean (SD)	Rapid decliners mean (SD)	P-value
N	77	90		107	150	
**Baseline Clinical Variables**						
Age	72.4 (7.6)	73.6 (7.8)		74.4 (7.6)	75.6 (6.6)	
Education	15.9 (2.9)	15.2 (2.6)		17.1 (2.4)	15.6 (3.2)	
APOE4+ (%)	38%	68%	p < 0.001	42%	59%	p < 0.01
ADAS-13	10.8 (4.5)	25.0 (3.6)		12.3 (2.9)	24.2 (4.3)	
MMSE	28.3 (1.5)	26.0 (1.3)	p < 0.001	28.0 (1.7)	26.7 (1.7)	p < 0.001
MOCA	25.1 (2.2)	20.2 (3.0)	p < 0.001	24.2 (2.7)	20.9 (2.5)	p < 0.001
RAVLT-%forgetting	43.5 (25.7)	82.6 (34.0)	p < 0.001	47.5 (27.7)	84.3 (23.1)	p < 0.001
FAQ	2.2 (5.0)	5.0 (3.5)	p < 0.001	2.2 (3.3)	5.5 (4.8)	p < 0.001
CDR-SB	1.4 (0.9)	2.0 (0.9)	p < 0.001	1.3 (0.8)	1.9 (1.0)	p < 0.001
**Baseline MRI, PET and CSF Variables**						
Ventricles	30 397 (14 872)	34 843 (14 144)	p < 0.05	44 956 (23 220)	53 591 (26 544)	p < 0.02
Hippocampus	6 664 (861)	5 798 (956)	p < 0.001	7 085 (861)	6 281 (992)	p < 0.001
Whole Brain	965 210 (90 020)	924 940 (88 040)	p < 0.01	1 057 130 (104 570)	1 045 230 (90 540)	
Entorhinal Cortex	3 510 (630)	2 854 (760)	p < 0.001	3 809 (713)	3 182 (658)	p < 0.001
Medial Temp	18 737 (2 537)	16 820 (2 531)	p < 0.001	20 447 (2 547)	19 310 (2 850)	p < 0.01
ABETA	174 (48)	145 (56)	p < 0.001	180 (57)	149 (46)	p < 0.02
T-TAU	87 (73)	133 (48)	p < 0.001	63 (73)	114 (63)	p < 0.001
P-TAU	34 (29)	53 (19)	p < 0.001	31 (18)	45 (20)	p < 0.001
FDG PET	1.27 (0.11)	1.15 (0.13)	p < 0.001	1.27 (0.11)	1.15 (0.13)	p < 0.001
Amyloid PET	1.17 (0.25)	1.39 (0.16)	p < 0.001	1.19 (0.19)	1.37 (0.24)	p < 0.01
**Longitudinal Clinical Data**						
SCDRSB	0.1 (0.7)	0.7 (0.5)	p < 0.001	0.0 (0.3)	0.7 (0.8)	p < 0.001
SADAS13	0.9 (2.7)	1.9 (1.8)	p < 0.001	0.6 (2.0)	1.8 (2.5)	p < 0.001
SMMSE	−0.3 (1.3)	−1.0 (0.8)	p < 0.001	−0.0 (0.7)	−1.1 (1.3)	p < 0.001
SFAQ	0.4 (2.2)	2.0 (1.2)	p < 0.001	0.5 (1.4)	1.6 (2.4)	p < 0.001
SMOCA	−0.1 (0.7)	−0.5 (0.7)	p < 0.01	−0.3 (1.1)	−0.8 (1.0)	p < 0.05
SRAVLT-immediate	−0.8 (3.3)	−1.6 (2.2)		−0.6 (2.5)	−1.4 (2.8)	p < 0.001
**Longitudinal MRI Slopes**						
SVentricles	1 120 (1 798)	2 091 (1 148)	p < 0.001	1 135 (1 029)	2 262 (1 813)	p < 0.001
SHippocampus	−66 (89)	−122 (80)	p < 0.001	−62 (72)	−108 (122)	p < 0.001
SWholeBrain	−3 410 (7 840)	−7 630 (5 380)	p < 0.001	−3 760 (6 460)	−7 560 (8 260)	p < 0.001
SEntorhinal Cortex	−30 (178)	−54 (136)	p < 0.02	−36 (203)	−81 (193)	p < 0.05
SFusiform	−94 (383)	−305 (242)	p < 0.001	−108 (353)	−268 (397)	p < 0.001
SMedial Temp	−159 (431)	−365 (286)	p < 0.001	−121 (289)	−331 (462)	p < 0.001

## Discussion

The early and accurate identification of subjects at risk for AD remains a priority for the field. Aging and AD both are well known to be heterogeneous conditions and decades of research have shown that individuals vary widely in many clinical, cognitive and pathological characteristics of brain aging^2–12^. Initial attempts to identify a homogenous group of individuals at risk for AD dementia led to the concept of MCI as a transition stage between aging and dementia^11, 12^. Subsequently, MCI was further subgrouped into amnestic, nonamnestic and multidomain types, and more recently into pathological subtypes based on amyloid scans or spinal fluid markers (reviewed in ref. 15). While these efforts have modestly improved the prognostic reliability, all of these classifications remain suboptimal since binary cut-off points for biomarkers remain insufficiently validated and many underlying processes may contribute to the heterogeneity of MCI and risk for AD.

The novelty of our work is the use of an unbiased clustering algorithm on baseline to identify clusters of slow and the rapid declining subjects within the category of late MCI. Our work (e.g. Table 2) clearly demonstrates that these subpopulations are markedly different in respect to baseline cognition, objectively measured baseline biomarker data, the rate of cognitive progression of these subjects and the rate of longitudinal brain atrophy. The rapid cluster had an almost 5-fold greater rate of converting to dementia versus the slow cluster and a lower rate of reverting back to cognitively normal state (0% versus 13%). The rapid cluster also had a 7-fold faster deterioration of global functioning as measured by the CDR-SB. The slope of the increase of the volume of lateral ventricles, a neuronal loss marker of disease progression, was almost 2-fold greater in rapid declining subjects versus slow declining patients. These data confirm that the rapid decliner subset of MCI is pathological different from the slow decliner subset – likely in a more advanced phase of the pathological process of AD - arguing for a need to further subclassify late MCI subjects.

From the correlation networks it can be noticed that values of biomarker data are more strongly correlated with slopes of changes of clinical scales than with their baseline values. This suggests that pathological biomarker changes precede the cognitive decline and supports their proposed causal role. Table 3 lists the mean and SD values for the Rapid Decliner and Slow Decliner group on a range of baseline characteristics. These results confirm the fact that baseline cognitive and biomarker status robustly predict cognitive decline in MCI but extends that to identify specific baseline cognitive, functional and biomarker characteristics that mark very slow and very rapid decliners. While baseline differences between these clusters are most significant in respect of ADAS scores they are apparent across a range of clinical and biomarker measures such as entorhinal cortex and hippocampal volume as well as amyloid and tau markers.

Using the data from all MCI subjects, we identified baseline classifiers that could predict rapid decline. We focused on cognitive tests since they are less expensive and more practical than PET scans or CSF data. We first tested the MMSE and found that even a conservative MMSE < 27 cutoff was a poor classifier for identifying rapid decliners. Currently subjects with MMSE score between 25–30 are routinely included in MCI clinical trials (as long as they meet other memory test score criteria) and our findings suggest this practice could be modified if there is a desire to identify rapid decliners. A combination of ADAS11 and 13 cut-offs proved the best classifier overall – with lower cut points than when these tests are applied individually. It had high specificity and sensitivity in our test sample (ADNI-1) with high reproducibility in the independent replication sample of ADNI-2 subjects. The differences between rapid and slow clusters (and unclassified patients) were very large for a number of markers thus explaining the high discriminatory effect. This is not totally surprising since prior studies have reported that baseline ADAS is a predictor of future decline. Because the ADAS-11 is part of the ADAS-13 test, MCI treatment trial sponsors could readily incorporate such a criterion for their studies to enrich their study for rapid decliners. While using such a cut-off would result in a need to screen more subjects and a higher screen fail rate, the robust decline of those enrolled could allow for more efficient trial design with smaller numbers of subjects randomized. The cut-offs listed in Table 3 would be useful both for sponsors planning for future clinical trials of therapies to prevent AD dementia as well as clinicians doing prognostic counseling of their patients.

There are several strengths to our report. To our knowledge, this is the first study to use multilayer cluster analyses to identify homogenous MCI clusters with diverse prognostic outcomes. An advantage of the unbiased methodology we use is that, in contrast to other approaches, we cluster cases that can be clustered and the rest remains simply unclustered. Clinically useful clustering should result in patient subpopulations that very similar with regards to multiple variables as documented by the large differences in multiple baseline and longitudinal variables between the rapid and slow clusters. ADNI is one of the most successful longitudinal biomarker studies in the AD field and its strengths include the use of more than 50 sites across the US, a prospective design, rigorous selection criteria for late MCI, careful standardization of clinical, imaging and biomarker data collection and longitudinal follow up for up to 5 years^21^. The late MCI subjects in ADNI-1 and ADNI-2 were recruited using identical criteria and hence provided an ideal test and validation sample for our identifying classifiers. We included the major baseline clinical, cognitive, activities of daily living and biomarker predictors that are routinely used since our goal was to make the findings relevant to clinical trials. There are also some limitations. ADNI subjects were recruited at leading academic research centers and while representative of subjects enrolled in clinical trials, may not be representative of all such subjects in the population. We restricted the follow up to 5 years to keep the maximum follow up period identical for ADNI1 and ADNI2 and hence we do not know if the outcomes would change with longer periods of follow up. Hence, the findings observed here, including the specificity and sensitivity of cut points to identify rapid decliners, need be replicated in larger population studies before they can be generalized.

Lastly, the identification of homogenous MCI subpopulations may also provide new insights into Alzheimer’s disease mechanisms. For example, it could be of interest to examine how the rapid and slow declining clusters differ in their genetic, transcriptomic, proteomic and metabolomics profiles. ApoE4 was overrepresented among rapid decliners but it does not explain all of the variability. The inclusion of baseline genomics data in such a cluster analyses could result in the identification of even more homogenous sets of MCI subgroups. One of the most important genetic subgrouping that has been relatively overlooked in the laboratory study of AD is the effect of gender – most rodent studies have been in male mice and only a small number of biomarker studies have tested for sex differences^6, 7^. Our analyses show that rapid MCI decliners in both sexes had worse baseline cognition, higher levels of cortical amyloid and tau pathology as well as smaller volumes of hippocampal and entorhinal cortices. This supports the use of these markers, in both men and women, to select at risk subjects in prevention trials. Further characterization of the genetic underpinnings of such divergent prognostic outcomes may potentially yield new prognostic tests for AD and novel biochemical targets for therapeutic drug discovery.

Clinical trials in MCI testing therapies to delay the onset of AD today require large sample sizes and long durations of follow up in order to achieve reliable rates of decline in the placebo groups^1, 2, 5, 14, 21^. This is because of the heterogeneous nature of MCI, the variables rates of conversion to AD from one subject to another and lack of an approved predictive test. Further, the fact that in such studies many patients will receive placebo for 18 months or longer raises the ethical dilemma of exposing subjects at risk of developing dementia to lengthy placebo treatment^22^. It is our hope that further research utilizing newer data mining approaches to identify clinically relevant subpopulations at risk for AD will not only accelerate the search for disease modifying therapies and development of prognostic tests but provide reassurance to those who may be at very low risk for progression.

## Materials and Methods

### Subjects

All protocols were approved by the Duke University Medical Center institutional review board and IRBs at each site (full list of all sites and IRBs is available at www.adni-info.org), and written informed consent was obtained from all subjects prior to enrollment. Data used in the preparation of this article were obtained from the Alzheimer’s Disease Neuroimaging Initiative (ADNI) database (adni.loni.usc.edu). The ADNI was launched in 2003 as a public-private partnership with a primary goal to test whether serial magnetic resonance imaging (MRI), positron emission tomography (PET), other biological markers, and clinical and neuropsychological assessment can be combined to measure the progression of mild cognitive impairment (MCI) and early Alzheimer’s disease (AD). ADNI (ADNI ClinicalTrials.gov identifier: NCT00106899) is the result of efforts of many coinvestigators from a broad range of academic institutions and private corporations, with subjects recruited from over 50 sites across the United States and Canada. Details of the ADNI-1 and ADNI-2 protocol, timelines, study procedures and biomarkers can be found in the ADNI-1 and ADNI-2 procedures manual [http://www.adni-info.org/]. For up-to-date information, see www.adni-info.org.

All ADNI-1 and ADNI-2 late MCI subjects with at least one post-baseline visit data were eligible for inclusion. The criteria for classification as late MCI in ADNI-1 and ADNI-2 are identical and are as follows: subjective memory complaint, objective evidence of impaired memory calculated by scores of the Wechsler Memory Scale Logical Memory II adjusted for education, absence of significant confounding conditions such as current major depressive episode, normal, or near normal daily activities, absence of clinical dementia, an inclusive mini-mental state examination (MMSE) score from 24–30, and a score of 0.5 on the global CDR. For a detailed list of all selection criteria, refer to the ADNI procedures manual [http://www.adni-info.org/]. In addition to demographic data, for subject inclusion, data for all the following parameters were required: Alzheimer’s Disease Assessment Scale-Cognitive subscale (ADAS-cog) for at least two different time points, genotyping results, and biomarker data at baseline. The term “baseline” is used to indicate data collected first at either screening or baseline. Additional details are provided in the ADNI procedures manual. Early MCI (EMCI) subjects were not included in this analysis.

### Clinical and Genetic Variables

Demographic variables included were age, gender, education level. APOE allele genotyping of all subjects was completed using DNA extracted from peripheral blood cells, with details provided elsewhere [http://www.adni-info.org]. In total, 378 MCI subjects from ADNI-1 were included. Cognitive and functional variables included were Alzheimer’s Disease Assessment Scale (ADAS-Cog 11 and 13), Mini Mental Scale Examination (MMSE), Montreal Cognitive Assessment (MOCA), and the Rey Auditory Verbal Learning (including subtests). Disease staging and activities of daily living scales included were the Clinical Dementia Rating (CDR-SB) and Functional Assessment Questionnaire (FAQ). Details of these tests can be found in the ADNI procedures manual [http://www.adni-info.org/].

### Pathological and Neuronal Loss Biomarkers

Imaging and spinal fluid data were downloaded from the ADNI dataset.

#### MRI Measures

Structural MRI brain scans were acquired using 3 T MRI scanners with a standardized protocol. Quantification was performed in an automated pipeline using FreeSurfer software package version 5.1 (http://surfer.nmr.mgh.harvard.edu/fswiki). Detailed descriptions can be found at www.adni-info.org. Volumetric or thickness data on whole brain, lateral ventricles, hippocampus, entorhinal cortex, fusiform and medial temporal lobe were included. These served as surrogate markers for neuronal loss. Intracranial volume was also included as a covariate. For more details of MR imaging procedure, readers are referred to http://adni.loni.usc.edu. Each brain volume indicated is a summation of right and left hemispheric region and the unit is in mm﻿^3^﻿.

#### FDG-PET Measures

18F-FDG-PET standardized protocols, acquisition and analyses methods are described at http://adni.loni.usc.eduqw/methods/pet-analysis/pre-processing/ and at http://www.adni-info.org/Scientists/ADNIStudyProcedures.html. Cerebral metabolic rate for glucose (CMRgl) values were analyzed. We classified FDG-PET as a metabolic marker rather than as a pathological marker by convention but acknowledge it can also mark neuonal injury and pathological changes.

#### Amyloid PET (Pathological biomarker)

18F-florbetapir brain PET (referred to as AV45 PET) measures fibrillary cortical amyloid deposition and global SUVr values were used for our analyses. The global summary measures relative cortical Aβ deposition in frontal, cingulate, lateral parietal, and temporal cortices. Methods used to acquire and process ADNI florbetapir PET image data can be found at http://adni.loni.usc.edu/methods/.

#### Cerebrospinal fluid (CSF) measures

CSF samples were obtained by lumbar puncture and examined for total tau, phosphorylated tau (p-tau_181P_), and amyloid-beta (Aβ_1–42_). CSF proteins were measured using the multiplex xMAP Luminex platform (Luminex Corp) with Innogenetics (INNO-BIA AlzBio3, for research use–only reagents) immunoassay kit–based reagents with details described elsewhere (www.adni-info.org).

MRI volumes, PET SUVRs and CSF protein levels were used as continuous variables.

ADNI-1 and ADNI-2 differed slightly in the numbers of subjects who had various biomarker tests. MRI was done in all subjects with at least one volumetric measure available in 561 subjects. CSF markers were available in 302 subjects (required for only a third of ADNI-1 subjects but required for all ADNI-2 subjects). FDG-PET was required for only half of ADNI-1 subjects and required for ADNI2 thus was available for 362 subjects. Florbetapir amyloid PET was done only in ADNI-2 and was available for 157 subjects. Details of imaging and spinal protein assay protocols, quality control and standardization across sites can be found on the ADNI website (http://www.adni-info.org/).

### Longitudinal Cognitive, Functional and MRI Data

MCI subjects were monitored in both ADNI-1 and ADNI-2 at 6 month intervals for up to 5 years. Cognitive and biomarker tests were administered at specific intervals. From the available longitudinal data we computed slopes for 10 clinical (SCDRSB, SADAS11, SADAS13, SMMSE, SFAQ, SMOCA, SRAVLTimmediate, SRAVLTlearning, SRAVLTforgetting, SRAVLTpercForgetting) and 7 imaging descriptors (SVentricles, SHippocampus, SWholeBrain, SEntorhinal, SFusiform, SMidTemp, SICV). Slopes are identified by the name of the corresponding baseline descriptor with added starting ‘S’. For example SFAQ denotes changes of FAQ. Its value is the mean increase or decrease in a 6 month period computed for the complete period in which the patient has been monitored. In the rest of paper the computed slopes are denoted as prognostic descriptors. ADNI-1 subjects were analyzed only through the end of ADNI-1 (first 5 years) to keep it comparable to newly recruited ADNI-2 subjects who were also followed for upto 5 years.

### Longitudinal Change in Diagnosis

The subject’s diagnosis was assessed at each visit by the site clinician using all available information. At each visit the MCI subject’s diagnosis could remain unchanged, or be changed to Dementia (if the subject worsened and met criteria) or be changed to Cognitively Normal (if the cognition had improved and subject no longer met MCI criteria). Subjects who met criteria for dementia were then further assessed to see if they met criteria for probable AD dementia. Details of criteria can be found in the ADNI procedures manual [http://www.adni-info.org/].

### Statistical and Data Mining Methods

#### Summary Statistics

For initial descriptive and slope analyses, we used standard statistical methods: Man-Whitney’s test to detect descriptors for which two populations are statistically different and Spearman’s correlation to detect pairs of related descriptors. Because of the large number of variables, non-parametric test was used to avoid assumptions about distributions of variables. Simple linear regression slopes, without any covariates, were computed for clinical and MRI variable of interest using all time points available. Non-parametric methods are used in order to avoid assumptions about distributions of descriptor values.

#### Correlation Network

For correlation network visualization *igraph* package in R was used to obtain Fruchterman-Reingold force-directed layout. Edges present Spearman’s rank correlations with value *rho* ≥ 0.*50*. Distance between nodes is defined as the inverse of the correlation: *dist* = *1/ rho*; small distance denotes large correlation. Fruchterman-Reingold force-directed graph technique was used to construct a layout in which strongly correlated concepts are next to each other and concepts that are strongly related to many other concepts are positioned in the center of the network. Baseline clinical and biomarker descriptors are denoted by squares while circles denote longitudinal slope descriptors. Green and yellow colors are used for clinical and MRI descriptors, respectively. Orange squares represent baseline 18F-FDG or 18F-florbetapir PET data while red squares are baseline spinal fluid data.

#### Multilayer Clustering Algorithms

A novelty of the work is application of a clustering tool for identification of homogeneous subpopulations of subjects. Although clustering is a well-known technique and many different algorithms are available, it is rarely used for insightful data analysis. The main reason is that application of different algorithms will typically result by different clusters. Each algorithm has parameters that have to be carefully adjusted by the user and whose selection also influences the final result^23^. In the absence of objective measures for the evaluation of the clustering results, a typical criterion for the selection of the most appropriate clustering algorithm and selection of its parameters is the usefulness of the clustering result^24^.

Multi-layer clustering algorithm has been used in this work because it enables the size and the number of clusters to be determined automatically. The algorithm consists of two steps; in the first step example similarity table (EST) is computed for each data layer and in the second step these tables are used by an agglomerative bottom-up procedure to find an optimal clustering solution. Similarity of instances is determined by execution of a supervised machine learning algorithm on an artificial classification task which is formulated so that original instances are positive class examples while randomized original instances are in the negative class^19^. The supervised learning algorithm constructs many rules that discriminate between original examples and randomized examples^20^. Similar positive examples are covered by many common rules while very different examples are rarely both covered by the same rule. EST is a symmetric NxN matrix where N is the number of original instances. Value in position *x*_*i, j*_ represents similarity of examples *i*, *j* which is computed as a proportion of rules that cover this pair of examples.

The second step of the multi-layer algorithm is a heuristic procedure aimed at finding an optimal solution in which each instance *i* is clustered together with all instances with which it has high similarity while instances with low similarity should stay outside this cluster. The Clustering Related Variability (CRV) score *CRV*_*i*_ is defined for each instance *i*

$$CR{V}_{i}=CR{V}_{i,wc}+CR{V}_{i,oc}$$

*CRV*_*i*, *wc*_ is within cluster variability while *CRV*_*i*,*oc*_ is outside cluster variability of EST values.

$$CR{V}_{i,wc}=\sum _{j\in C}{({x}_{i,j}-{x}_{mean,wc})}^{2}$$

$$CR{V}_{i,oc}=\sum _{j\notin C}{({x}_{i,j}-{x}_{mean,oc})}^{2}$$

*CRV*_*i*, *wc*_ is computed from the values that are in row *i* and those columns corresponding to instances that are in the same cluster *C* as the instance *i*. Value *x*_*mean*, *wc*_ is the mean value of *x*_*i, j*_ in cluster C while value *x*_*mean*, *oc*_ is the mean value for all other *x*_*i*, *j*_ values in the row *i*. If example *i* is the only one example in cluster *C* then *CRV*_*i*, *wc*_ = 0 and *CRV*_*i*, *oc*_ is equal to the variability of all *x*_*i*, *j*_, *i* ≠ *j*. Clustering related variability for a cluster *C*, $CR{V}_{C}={\sum }_{i\in C}CR{V}_{i}$ is defined as a sum of *CRV*_*i*_ values for all instances included into the cluster. For each pair of clusters *x*, *y* the value

$$DIF{F}_{xy}=CR{V}_{x}+CR{V}_{xy}-CR{V}_{xy}$$

can be computed. *DIFF*_*xy*_ has a positive value if merging clusters *x* and *y* enables reduction of the clustering related variability. In multi-layer clustering when two data layers are defined then EST and *DIFF*_*xy*_ are computed independently for each data layer. In this case the joint *DIFF*_*xy*_ is the smaller one of differences for both layers:

$$DIF{F}_{xy}=\,\min (DIF{F}_{xy,layer1},DIF{F}_{xy,layer2}).$$

The goal is to find a clustering solution so that for all constructed clusters the clustering related variability *CRV*_*C*_ is minimal. The clustering starts with each example in its own cluster. In every iteration *DIFF*_*xy*_ is computed for all possible pairs of clusters *x*, *y* in the current solution and the pair with maximal *DIFF*_*xy*_ is selected. If this maximal value is positive it means that further reduction of variability is possible. Clusters *x* and *y* are merged and the next iteration starts. Otherwise, clustering procedure ends with the current solution as the optimal one. Details of the algorithm have been published previously^25, 26^.

The multi-layer algorithm is the substantial part of the web application called Exploratory Clustering. It is publicly available at http://rr.irb.hr/exploC/ ^27^. The tool can be used for various clustering tasks with up to 1000 instances and 1000 attributes. The ADNI baseline data for all MCI patients are loaded into the first data layer, and the second data layer consists of slopes of values computed from longitudinal data. In all, 26 baseline and 17 longitudinal variables were input. The tool is unbiased and clusters patients based on their variables and then based on the properties of obtained clusters the user can distinguish different clinically relevant subpopulations. The difference among experiments is that various subsets of input attributes are used for the computation of the similarity of instances.

#### Identifying and Validating Classifiers

Subgroup discovery technique was used to identify the best classifiers (clinical test cut-offs on ADAS, MMSE and RAVLT) to identify MCI rapid decliners as well as to compute sensitivity and specificity of constructed classifiers. The classifiers were first developed using ADNI-1 study MCI data and then replicated and validated using the ADNI-2 study MCI data. All MCI subjects were included in this analysis including slow and unclassified subgroups.

All methods were performed in accordance with the relevant ethical guidelines and regulations as as stated in the first section of Methods.
